# Application of a modified Peyton’s four-step approach in clinical skills training for residents: a randomized controlled trial

**DOI:** 10.1186/s12909-026-09499-8

**Published:** 2026-05-20

**Authors:** Bingyan Liu, Shufang Xiang, Baolai Xiao, Jinjian Xiang, Yimin Sun, Yuan Ren

**Affiliations:** 1 Department of Otorhinolaryngology-Head and Neck Surgery, The First Affiliated Hospital of Yangtze University, Jingzhou, China; 2 Department of Ultrasound, The First Affiliated Hospital of Yangtze University, Jingzhou, China; 3 Department of Gastrointestinal Surgery, The First Affiliated Hospital of Yangtze University, Jingzhou, China; 4 Department of Chemoradiotherapy, The First Affiliated Hospital of Yangtze University, Jingzhou, China

**Keywords:** Clinical skills, Standardized residency training, Peyton's four-step teaching approach, Video recording

## Abstract

**Background:**

Peyton’s four-step approach is a common teaching method in medical resident training. However, it has shortcomings such as long teaching duration, low efficiency, and poor long-term retention. This study proposed a modified Peyton’s four-step approach and explored its effectiveness in teaching clinical skills for wound dressing and suture removal to residents.

**Methods:**

One hundred and twenty residents were selected and randomly divided into an experimental group (*n* = 60) and a control group (*n* = 60). The experimental group received training using the modified Peyton’s four-step approach, while the control group received training using the traditional Peyton’s four-step approach. Teaching outcomes were evaluated by assessing residents’ clinical skills through objective structured clinical examinations (OSCEs) and administering questionnaires immediately after training and one month later.

**Results:**

After training, the OSCE scores of the experimental group (94.80 ± 3.02) were higher than those of the control group (93.15 ± 2.62). Notably, one month after training, the OSCE scores of the experimental group (94.13 ± 2.00) remained significantly higher than those of the control group (88.30 ± 4.89). These differences were statistically significant (*p* < 0.05). Questionnaire surveys indicated that residents in the experimental group believed the modified Peyton’s four-step approach better stimulated learning interest, improved active learning, enhanced teamwork, and strengthened clinical skills. Satisfaction with the teaching method was also higher in the experimental group compared to the control group (*p* < 0.05).

**Conclusion:**

The modified Peyton’s four-step approach demonstrates significant teaching effectiveness in the clinical skills training of residents. It not only improves the level of clinical operational skills but also enhances teaching efficiency and short- to medium-term skill retention, making it a superior teaching model worthy of promotion.

## Introduction

Standardized Residency Training (SRT) is an essential pathway for resident physicians transitioning into clinical practice. Clinical skills training plays a pivotal role in SRT, serving not only to equip residents with proficient operational abilities but also as a criterion for assessing their competence in actual clinical work [1, 2]. Although the traditional Peyton’s four-step approach provides a structured framework for clinical skills teaching, it suffers from long teaching duration, low instructional efficiency, and poor long-term skill retention, which limit its application in time-restricted residency training programs. In recent years, some scholars have introduced and modified the Peyton teaching approach, achieving favorable educational outcomes [3–7].

The Peyton teaching approach is an instructional method for clinical skills training, initially proposed by European scholars Walker and Peyton [8]. This method employs four steps to teach clinical skills:

- Step 1 - Demonstration: The instructor performs the skill at normal speed without explanation while the student observes.
- Step 2 - Deconstruction: The instructor repeats the performance, breaking it down into manageable segments and explaining the key points of each step.
- Step 3 - Comprehension: The student talks the instructor through the procedure, describing each step. The instructor provides feedback on the accuracy of the student’s instructions.
- Step 4 - Performance: The student independently performs the entire procedure under the instructor's supervision until proficiency is achieved [9].

In the field of medical education, the traditional Peyton’s four-step approach has been widely utilized for clinical skills training [10, 11]. However, with evolving educational demands, its limitations have become increasingly apparent [12, 13]. The traditional method presents several notable challenges. Firstly, it is relatively time-consuming, occupying significant instructor time and potentially limiting the diversity of teaching activities within constrained training schedules [14]. Secondly, while students often demonstrate adequate acquisition immediately following training, knowledge and skill retention can decline over time, leading to the recurrence of previously addressed errors [15]. This impacts the continuity of learning and diminishes the long-term effectiveness of training.

To address these shortcomings, this study innovatively incorporates structured peer-assisted learning and a video recording component into the foundational Peyton’s four-step model. This modified approach not only reduces direct instruction time but also provides significant convenience for post-session review. By revisiting the recorded videos, students can reinforce their memory of procedural steps and key concepts, thereby mitigating skill decay. Furthermore, these resources serve as a readily available reference for troubleshooting recurring problems, helping to prevent the repetition of mistakes [16]. This innovative teaching model holds promise for effectively enhancing the outcomes of clinical skills training for residents, laying a solid foundation for cultivating more highly competent medical professionals.

## Methods

### Design and participants

This study employed a prospective, single-center, randomized controlled design. The protocol was approved by the hospital’s Ethical Review Board. A total of 120 resident physicians who underwent standardized residency training between January 2023 and June 2024 were enrolled. Written informed consent was obtained from all participants prior to enrollment. The inclusion criteria were as follows: (1) good communication and comprehension skills; (2) full compliance with the curriculum schedule, with no absences or skipped sessions; and (3) completion of all training assignments. Exclusion criteria included: (1) non-compliance with the curriculum schedule; (2) inability to complete the training sessions; and (3) engagement in examination misconduct. Communication and comprehension skills were assessed by the resident physicians’ supervising attending physicians during routine clinical work in the two weeks prior to enrollment, using a simple three-point scale (good/qualified/poor); only those rated as “good” were included in the study.

### Intervention

#### Control group instruction

The 60 residents in the control group were randomly and equally divided into 4 subgroups. Instructors used the traditional Peyton’s Four-Step Teaching Approach to train the four subgroups in wound dressing and suture removal skills in batches. The training time for each subgroup was recorded. The specific implementation steps are detailed in Table 1.

**Table 1:** Detailed implementation methods of traditional vs. modified Peyton’s four-step teaching approach

Traditional Peyton’s Four-Step Teaching Method	Modified Peyton’s Four-Step Teaching Method
Demonstration: The instructor explains and performs a demonstration without any further explanation.	The instructor plays a video of wound dressing and suture removal without any narration. Residents watch the video while simultaneously observing, learning, and thinking.
Deconstruction: The instructor repeats the wound dressing and suture removal procedure, breaking it down into steps, demonstrating and explaining the key points of each step. Residents do not ask questions.	The instructor deconstructs the wound dressing and suture removal procedure into steps and provides a detailed explanation of each step to ensure residents fully understand and grasp the essentials. Residents do not ask questions during the explanation; they pose questions after the explanation is completed, and the instructor provides detailed answers.
Comprehension: The resident describes and explains each specific step of the procedure. The instructor performs the actions and provides feedback on the accuracy of the resident’s description/instructions.	Residents are divided into groups of three: ① Group A verbalizes the steps, Group B performs the demonstration, and Group C scores the performance and records identified issues. Roles are rotated. ② The issues are consolidated, and one designated resident feeds them back to the instructor. The instructor then addresses each raised question.
Demonstration: The resident independently practices the wound dressing and suture removal procedure. The instructor provides on-site supervision and guidance until proficiency is achieved.	Residents independently practice and are assessed on the wound dressing and suture removal procedure (no commentary is provided during the performance). The session is video recorded. Upon completion, the instructor provides immediate feedback and scores the performance. After the session, residents review the video content for self-directed learning.

#### Experimental group instruction

**Fig. 1:**
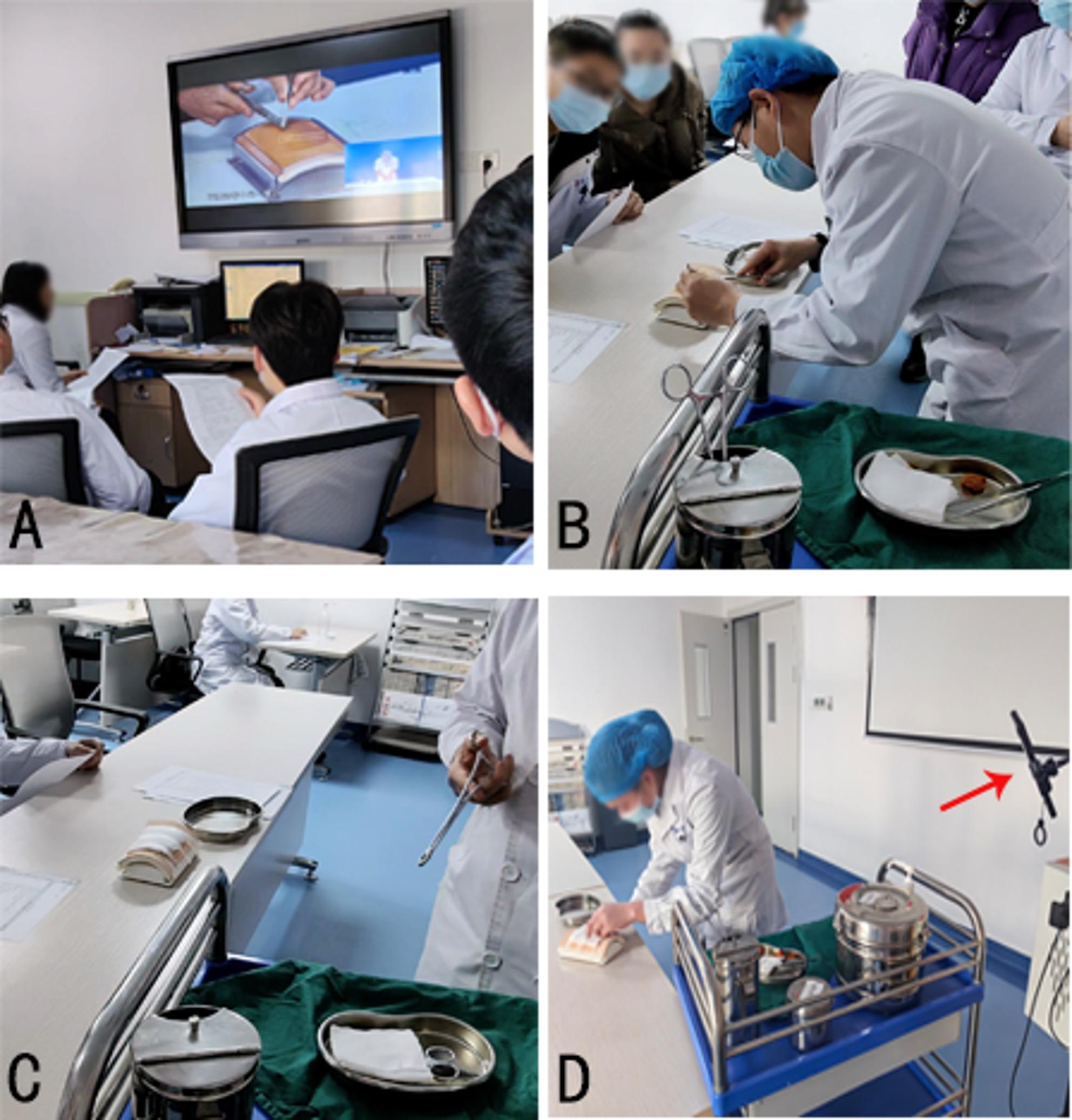
Modified Peyton’s four-step teaching model. **A** Video playback. **B** Deconstruction. **C** Peer learning and mutual supervision. **D** Demonstration with video recording (indicated by red arrows)

The 60 residents in the experimental group were randomly and equally divided into 4 subgroups. Instructors, who had undergone pre-service standardized training to achieve proficiency in the specific operational steps and workflow of the modified Peyton’s Four-Step Teaching Method, subsequently conducted the wound dressing and suture removal skills training for the four subgroups in batches. The total training duration for each subgroup (from the start of Step 1 to the completion of Step 4) was recorded by an independent research assistant using a stopwatch. The specific implementation steps are presented in Table 1; Fig. 1.

#### Randomization and blinding

Simple randomization was performed using SPSS software. Allocation concealment was achieved through the sealed envelope method, whereby randomly generated training protocols for each subgroup were placed into opaque, sealed envelopes. The envelopes were opened immediately prior to the training sessions, and the corresponding protocol was implemented. Outcome assessors and the statistician were blinded to group assignment. Due to the nature of the interventions, neither the instructors nor the resident participants could be blinded to the teaching method used.

### Outcomes

#### Clinical skills assessment

Residents from both groups were assessed on wound dressing and suture removal skills before the training, immediately after, and one month after the intervention. The assessment consisted of 11 items: proper preparation of materials, aseptic technique, correct disinfection area, appropriate number of disinfection passes, proper handling of forceps, correct transfer of items, accurate cutting of sutures, proper traction on suture ends, appropriate application of dressings, correct disposal of waste materials, and adequate patient instruction. A 100-point scoring system aligned with the training syllabus was used for evaluation. Two consistent examiners (physicians with senior professional titles) scored all participants using the same standardized criteria. Composite scores were calculated as the mean across the multiple assessment items.

#### Questionnaire survey

One month after the training, a satisfaction questionnaire on the teaching effectiveness was administered to both groups. The questionnaires were distributed and collected on-site. The survey dimensions included: stimulating learning interest, improving clinical skills, promoting active learning, enhancing teamwork, and overall satisfaction. Satisfaction levels were rated on a 5-point Likert scale: Very satisfied (5 points), Satisfied (4 points), Moderately satisfied (3 points), Dissatisfied (2 points), and Very dissatisfied (1 point).

#### Follow-up

At 1-month post-training, a clinical skills assessment and a teaching satisfaction questionnaire were administered to trainees in both groups. All outcome assessments were performed independently by physicians not involved in the research team.

#### Sample size

A preliminary experiment involving 10 participants in the experimental group (mean score = 94.70, SD = 2.87) and 10 participants in the control group (mean score = 93.00, SD = 3.37) provided initial data for sample size estimation. Using G*Power software (Version 3.1.9.7) with a significance level (α) of 0.05 for a two-tailed test and a statistical power target of 80%, the initial calculation indicated that 54 participants per group would be required to detect statistically significant differences. Accounting for an anticipated 10% dropout rate, this study plans to enroll a total of 120 participants (60 per group) to ensure adequate statistical power and robustness of the findings.

### Statistical analysis

Data are presented as mean ± standard deviation (normally distributed), median (range) (non-normally distributed), or frequency (percentage) (categorical variables). Intergroup comparisons were performed using the t-test, the Mann-Whitney U test, and the chi-square/Fisher’s exact test, respectively. Univariate and multivariate logistic regression analyses were employed to identify independent factors influencing training effectiveness. A two-tailed P value < 0.05 was considered statistically significant. All statistical analyses were conducted using SPSS 27.0, and graphs were generated with GraphPad Prism 9.0.

## Results

**Fig. 2:**
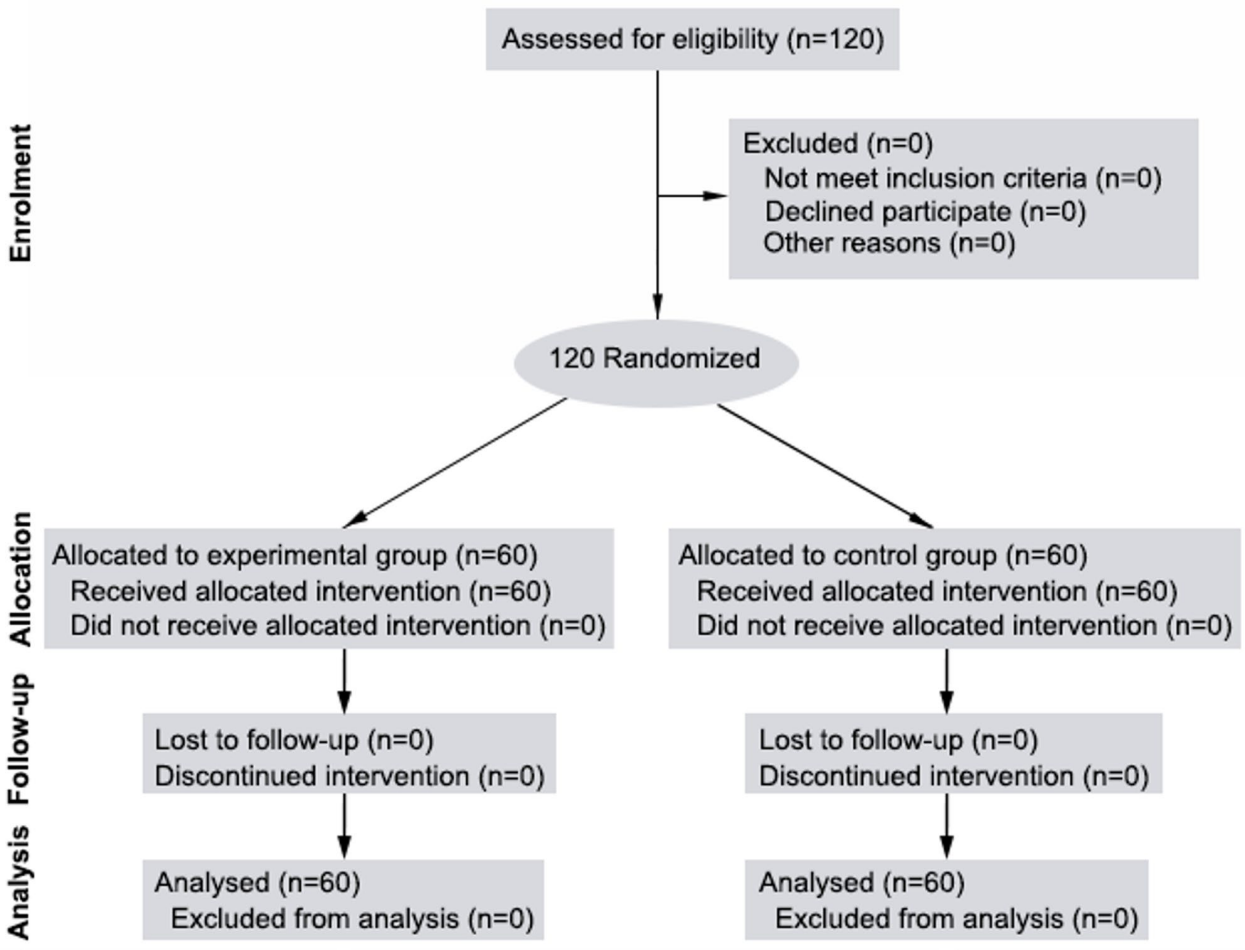
Flow diagram

This study enrolled a total of 120 eligible participants. During the course of the investigation, none of the enrolled residents withdrew or were lost to follow-up (Fig. 2). As shown in Table 2, there were no statistically significant differences between the two groups in terms of demographic characteristics, years of residency training, educational background, residency specialty, or surgical clinical experience.

**Table 2:** Baseline characteristics of participants

Index	Experimental group	Control group	*P*
Gender			0.854
Male	33 (55.0%)	34 (56.7%)	
Female	27 (45.0%)	26 (43.3%)	
Age (years)	25.7 ± 1.58	25.9 ± 1.57	0.488
Residency Duration (Years)			0.465
1	32 (53.3%)	28 (46.7%)	
2	28 (46.7%)	32(53.3%)	
Handedness			1.000
Right	60 (100%)	60 (100%)	
Left	0	0	
Degree			0.803
Bachelor’s Degree	51 (85.0%)	50 (83.3%)	
Master’s Degree	9 (15.0%)	10 (16.7%)	
Specialty			1.000
Internal Medicine	28 (46.7%)	28 (46.7%)	
Surgery	32 (53.3%)	32 (53.3%)	
Surgical Clinical Experience			0.729
No	56 (93.3%)	55 (91.7%)	
Yes	4 (6.7%)	5 (8.3%)	

### Primary outcome

#### Clinical skills assessment upon completion of training

There were no significant differences in the 11 assessed operational tasks between the experimental and control groups immediately after training. However, at the one-month follow-up, the experimental group demonstrated significantly higher scores in six specific tasks: correct preparation of materials, aseptic technique, appropriate disinfection area, proper handling of forceps, correct passing of items, and adequately informing patients of precautions (all *P* < 0.05; Table 3 and 4). After training, the experimental group had higher total clinical skills assessment scores than the control group, and this superiority was particularly pronounced at the one-month follow-up, with a statistically significant difference (*P* < 0.05; Table 5).

**Table 3:** Participant performance in the experimental vs. control group

Checklist item	Pre-course test			Post-course test		
	Experimental group (*n* = 60)	Control group (*n* = 60)	*P*	Experimental group (*n* = 60)	Control group (*n* = 60)	*P*
Preparation of sterile items ^a^	10 (16.7%)	11 (18.3%)	0.810	55 (91.7%)	52 (86.7%)	0.378
Aseptic operation ^a^	5 (8.3%)	4 (6.7%)	0.729	50 (83.3%)	45 (75.0%)	0.261
Scope of disinfection ^a^	47 (78.3%)	50 (83.3%)	0.487	56 (93.3%)	55 (91.7%)	0.729
Number of disinfection ^a^	52 (86.7%)	50 (83.3%)	0.609	58 (96.7%)	58 (96.7%)	1.000
Holds forceps ^a^	43 (71.7%)	45 (75.0%)	0.680	53 (88.3%)	50 (83.3%)	0.432
Pass items ^a^	27 (45.0%)	26 (43.3%)	0.854	54 (90.0%)	50 (83.3%)	0.283
Cut off the line knot ^a^	53 (88.3%)	53 (88.3%)	1.000	58 (96.7%)	58 (96.7%)	1.000
Traction on the Suture Knot ^a^	50 (83.3%)	54 (90.0%)	0.283	58 (96.7%)	58 (96.7%)	1.000
Cover sterile dressings ^a^	47 (78.3%)	45 (75.0%)	0.666	58 (96.7%)	57 (95.0%)	0.648
Dispose of medical waste ^a^	35 (58.3%)	37 (61.7%)	0.709	55 (91.7%)	55 (91.7%)	1.000
Patient education	25 (41.7%)	26 (43.3%)	0.853	55 (91.7%)	54 (90.0%)	0.752

^a^ N (%) of participants who correctly demonstrated the checklist item

**Table 4:** Participant performance in the experimental vs. control group

Checklist item	Post-course test (1-month)		
	Experimental group (*n* = 60)	Control group (*n* = 60)	*P*
Preparation of sterile items ^a^	56 (93.3%)	45 (75.0%)	0.006
Aseptic operation ^a^	52 (86.7%)	40 (66.7.0%)	0.010
Scope of disinfection ^a^	57 (95.0%)	50 (83.3%)	0.040
Number of disinfection ^a^	58 (96.7%)	57 (95.0%)	0.648
Holds forceps ^a^	55 (91.7%)	46 (76.7%)	0.024
Pass items ^a^	55 (91.7%)	45 (75.0%)	0.014
Cut off the line knot ^a^	58 (96.7%)	56 (93.3%)	0.402
Traction on the Suture Knot ^a^	58 (96.7%)	56 (93.3%)	0.402
Cover sterile dressings ^a^	57 (95.0%)	53 (88.3%)	0.186
Dispose of medical waste ^a^	56 (93.3%)	50 (83.3%)	0.088
Patient education	57 (95.0%)	50 (83.3%)	0.040

^a^ N (%) of participants who correctly demonstrated the checklist item

**Table 5:** Comparison of operational skills assessment results between the two groups (Mean ± SD, points)

Group	Pre-course test	Post-course test	Post-course test (1-month)
Experimental group	53.97 ± 7.08	94.80 ± 3.02	94.13 ± 2.00
Control group	54.23 ± 5.56	93.15 ± 2.62	88.30 ± 4.89
*t*	−0.230	3.20	8.551
*P*	0.819	0.002	< 0.001

### Secondary outcomes

**Fig. 3:**
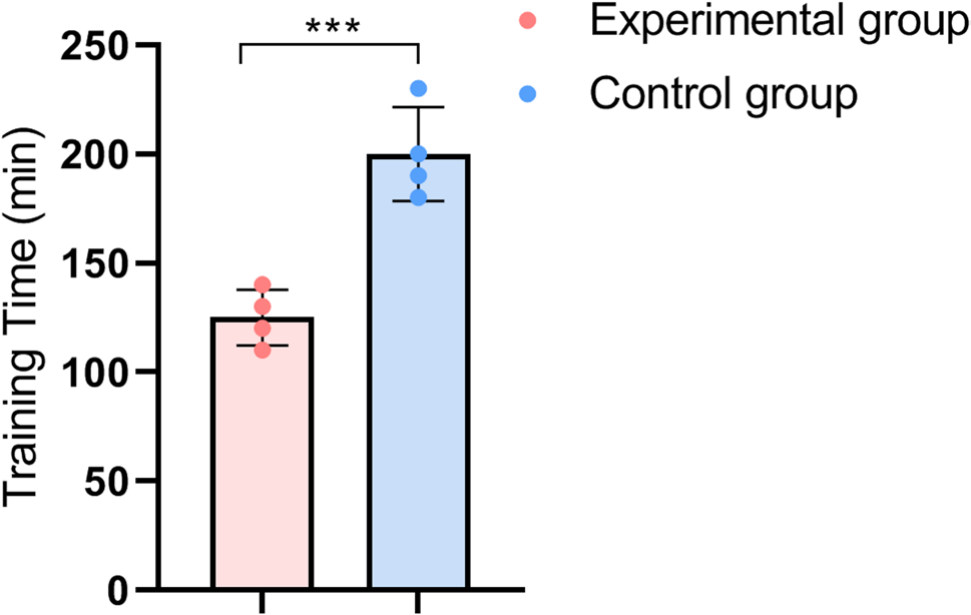
Comparison of training time between the experimental and control groups. ***P < 0.001

There was a significant difference in training time between the two groups. The experimental group demonstrated a significantly shorter training time, with a mean of 125 min (SD = 12.9 min), compared to 200 min (SD = 21.6 min) in the control group (*P* < 0.001; Fig. 3).

A comparison of teaching satisfaction was conducted between the two groups of residents. The survey results demonstrated that the overall satisfaction was significantly higher in the experimental group than in the control group, particularly in areas of stimulating learning interest, improving clinical skills, stimulating active learning, and enhancing teamwork (*P* < 0.05; Table 6).

**Table 6:** Comparison of teaching satisfaction between two groups (Mean ± SD)

Item	Experimental group (*n* = 60)	Control group (*n* = 60)	t	*P*
Stimulate learning interest	4.27 ± 0.58	4.05 ± 0.43	2.331	0.022
Improve clinical skills	4.63 ± 0.52	3.23 ± 0.93	10.202	< 0.001
Stimulate active learning	4.52 ± 0.54	3.52 ± 0.62	9.411	< 0.001
Enhance teamwork	4.33 ± 0.57	4.10 ± 0.48	2.427	0.017
Overall satisfaction	4.50 ± 0.50	3.38 ± 0.80	9.110	< 0.001

## Discussion

In this study, we compared a modified Peyton’s four-step teaching method (incorporating group peer learning, mutual supervision, centralized feedback, and smartphone video recording) with the traditional Peyton method for clinical skills training in resident physicians. The modified approach resulted in significantly higher operational skill assessment scores at the end of training, and this advantage remained statistically significant at the one-month follow-up. Furthermore, the total training time was markedly shorter in the experimental group, and residents in this group reported higher satisfaction in terms of stimulated learning interest, enhanced teamwork, and improved active learning. These findings are important for the medical education literature because they demonstrate that a structured, resource-efficient modification of a well-established teaching framework can simultaneously improve both short-term skill acquisition and short-to-medium-term retention while reducing instructor time-a critical consideration in high-demand clinical training environments where teaching resources are often limited.

The superior outcomes observed in the experimental group can be attributed to several components of the modified method. The integration of group peer learning, mutual supervision, and centralized feedback, together with the use of smartphone video recordings, appears to have reinforced the learning process. Video recording offers advantages such as convenience, storability, replayability, and ease of identifying errors, enabling residents to review and consolidate their skills during spare moments, a finding consistent with previous research [17]. Moreover, the “group peer learning, mutual supervision, centralized feedback” model adopted in the third step replaced the traditional one-on-one teacher-student instruction. This shift liberated instructors from repetitive demonstrations, while residents, by assuming the roles of both “instructor” and “examiner,” enhanced their self-directed learning and collaborative abilities, thereby improving overall teaching efficiency [18].

Questionnaire results showed that all residents in the experimental group reviewed their own video recordings prior to the one-month assessment. Residents in the experimental group rated higher than those in the control group in terms of stimulated learning interest and enhanced teamwork skills, with particularly notable advantages in improving clinical skills and promoting active learning. These findings may be attributed to the introduction of the “video playback” and “group peer learning, mutual supervision” mechanisms in the modified method, which disrupt the traditional teacher-centered instructional model, increase the novelty and engagement of the teaching format, and consequently improve resident satisfaction with the teaching. This is consistent with previous literature [19].

This study has several limitations. Although the sample size met the statistical requirements, large-scale, multicenter, and long-term studies are still needed to further validate the generalizability of our findings. Blinding was implemented for outcome assessors and data analysts; however, due to the observable nature of the interventions (e.g., video recording, group interactions), complete blinding of the instructors and residents was not feasible. This may have introduced performance bias, particularly affecting the results of the satisfaction assessments. Furthermore, this study only compared the modified Peyton teaching method with the traditional Peyton approach and did not include a control group using other modern teaching methods (e.g., simulation-based training, virtual reality instruction). Therefore, a comprehensive evaluation of the relative advantages of the modified method could not be conducted. In addition, satisfaction was assessed using a five-point Likert scale, which, although simple and practical, has not been validated for reliability and validity. Future studies could employ validated assessment tools, such as the visual analog scale, to enhance the credibility of the results.

## Conclusion

This study preliminarily explored the application of the modified Peyton four-step teaching method in clinical skills instruction for resident physicians. Compared with the traditional Peyton teaching method, this model not only improved residents’ procedural skills in a shorter period of time but also demonstrated better short-to medium-term skill retention. Simultaneously, it helped stimulate learning interest, improve clinical skills, promote active learning, and enhance teamwork abilities, receiving widespread acceptance among resident physicians. This teaching method shows promising application prospects and warrants further exploration and promotion within broader contexts.

## Data Availability

The datasets generated and analyzed during this study are available from the corresponding author upon reasonable request.
